# Association of Plant-Based and High-Protein Diets with a Lower Obesity Risk Defined by Fat Mass in Middle-Aged and Elderly Persons with a High Genetic Risk of Obesity

**DOI:** 10.3390/nu15041063

**Published:** 2023-02-20

**Authors:** James W. Daily, Sunmin Park

**Affiliations:** 1 Department of R & D, Daily Manufacturing Inc., Rockwell, NC 28138, USA; 2 Department of Food & Nutrition, Obesity/Diabetes Center, Hoseo University, Asan 31499, Republic of Korea

**Keywords:** BF, polygenic risk scores, plant-based diet, vitamin C, vitamin D, fiber

## Abstract

Obesity has become a severe public health challenge globally. The present study aimed to identify separate and interactive dietary, genetic, and other factors that increase the risk of obesity as measured by body fat (BF) mass. We utilized a genome-wide association study to identify genetic variants associated with high fat mass (obesity; *n* = 10,502) and combined them to generate polygenic risk scores (PRS) of genetic variants interacting with each other in adults aged over 40 while excluding body-fat-related diseases in a city-hospital-based cohort (*n* = 53,828). It was validated in Ansan/Ansung plus rural cohorts (*n* = 13,007). We then evaluated dietary and lifestyle factors in subjects to assess what factors might help overcome a genetic propensity for higher BF. The three-SNP model included brain-derived neurotrophic factor (*BDNF*)_rs6265, fat-mass- and obesity-associated protein (*FTO*)_rs1421085, and *SEC16B*_rs509325. The genes with the minor alleles of *ADCY3*_rs6545790 and *BAIAP2*_rs35867081 increased their gene expression in the visceral and subcutaneous adipocytes, but their gene expression decreased in the hypothalamus in eQTL analysis. In the three-SNP model, the PRS was associated with BF mass by 1.408 and 1.396 times after adjusting covariates 1 (age, gender, survey year, residence area, education, and income) and 2 (covariates in model 1 plus energy intake, alcohol intake, regular exercise, and smoking status), respectively. However, when separating subjects by PRS of the three-SNP model, a plant-based diet was the most significant factor associated with low BF, followed by high-protein diets and lower energy intakes. They could offset the effects of high genetic risk for high BF. In conclusion, modulating nutrient intakes might overcome a high genetic risk for obesity. Dietary choices favoring more plant-based and higher-protein foods might help prevent increased BF in Asians and potentially people of other ethnicities with high polygenetic risk scores.

## 1. Introduction

Obesity is a multi-factored pathological condition involving genetic, environmental, and behavioral factors. Despite intense research efforts over many years, no practical solutions for the prevention and treatment of obesity have been realized. It has been estimated that 40–75% of the variability in body mass index (BMI) is genetic [1]. However, known combinations of single-nucleotide polymorphisms (SNPs) can only predict about 6% of the BMI variability in people of European descent [2]. Although genetic susceptibility to obesity is incompletely described, genome-wide association studies (GWAS) have identified patterns of genetic variations that increase the risk of obesity [3]. Applicability of these genetic factors among all populations is impossible since there are ethnic differences in the risk alleles [4], and many study results may not apply to people of different races. It is also unknown whether genetic predispositions to obesity play a causative or permissive role in most obesity. There have always been examples of people with severe obesity due to inherited traits, but that is uncommon. The recent rapid global increases in obesity rates suggest a more permissive role since the genomes of populations are slow to evolve [5]. It is well known that many people who carry obesity-related genes do not become obese, and for most people, genetics do not equate to destiny [5]. Genetic traits also interact with lifestyles, including dietary patterns. Dietary patterns include a Korean balanced diet (KBD), plant-based diet (PBD), Western-style diet (WSD), and rice-main diet (RMD) in Korea. Their intakes influence obesity differently when interacting with a genetic predisposition [6].

Many studies use BMI as a measure of obesity, but BMI may not represent obesity equally in people, especially in those with a high muscle mass or different fat distributions. It has been demonstrated that people of different ethnicities with similar BMI may exhibit rather significant differences in fat mass [7]. BMI reflects lean body mass and fat mass, but obesity is best defined by fat mass, which is difficult to measure accurately. Furthermore, separating subcutaneous fat from visceral fat is difficult. However, body fat mass can be determined by dual-energy X-ray absorptiometry (DEXA), bioelectrical impedance analysis (BIA), and hydrodensitometry. DEXA and hydrodensitometry can estimate body fat more accurately than BIA [8], and DEXA can predict visceral and subcutaneous fat mass separately. However, DEXA and hydrodensitometry may not be applicable to epidemiological studies [9]. The BIA method is substantially accurate for measuring body fat mass. Therefore, we conducted a GWAS study in a large Korean cohort and evaluated dietary and lifestyle factors that interact with genetic predispositions to obesity defined by body fat mass measured by the BIA method.

The association of fat mass with genetics needs to be studied in large cohorts since numerous dietary and lifestyle factors make small but statistically significant contributions to fat mass. In the Korean Genome and Epidemiology Study (KoGES), fat mass was measured in the Ansan/Ansung cohort (*n* = 8842) by BIA and estimated in a city-hospital-based cohort study (*n* = 58,701) by a prediction model generated by a machine learning approach. In an attempt to isolate more significant effects in real-life interventions, we took the analysis a step further and developed polygenic risk scores (PRS) for obesity and separated the population according to those into high and low PRSs and then divided each into those with high and low body fat composition. We hypothesized that investigating people with a high PRS that do not develop obesity would provide information on the most important dietary factors required to maintain a lean body type even in those predisposed to obesity. This study aimed to determine the fundamental approaches to avoiding obesity in the Korean population.

## 2. Methods

### 2.1. Participants and Setting

The institutional review board (IRB) of the Korean National Institute of Health and the IRB of Hoseo University approved the KoGES and the present study (KBP-2015-055 and 1041231-150811-HR-034-01, respectively). All participants signed a written informed consent form. In this study, 58,701 participants aged 40–74 years were recruited from a large city-hospital-based cohort that formed part of the Korean Genome and Epidemiology Study (KoGES), which was conducted during 2010–2014 [10]. Adults aged over 40 (*n* = 13598) that participated in the Ansan/Ansung plus rural cohorts during 2001–2002 were used as the replicate study for exploring fat-mass-related genetic variants. People with a disease history that might influence energy metabolisms, such as cancers, thyroid diseases, chronic kidney disease, and brain-related diseases, were excluded from the study. The 4873 and 591 participants were excluded from the urban-hospital-based cohort and Ansan/Ansung plus rural cohorts, respectively. The participants included in the further study were 53,828 for the city-hospital-based cohort and 13,007 for the Ansan/Ansung plus rural cohorts for the replicate study.

### 2.2. Demographic, Anthropometric, and Biochemical Parameters of the Participants

The information on age, gender, education, income, alcohol and coffee consumption, physical activity, and smoking history was received during a health interview. Alcohol intake was calculated as the daily amount (g/day) by multiplying the frequency of drinking and the amount at an event [11]. The smoking status was categorized into current smokers (smoked at least 20 cigarettes in the past six months), past smokers (not smoking for at least six months), and non-smokers [11]. The coffee intake was assessed as the weekly drinking frequency and was categorized into three groups by the tertiles of daily coffee intake. Regular physical activity was defined as more than 30 min of moderate physical activity for three or more days per week.

The anthropometric characteristics (height and weight) were measured at the initial visit, as described previously [11]. Body fat and skeletal muscle masses were determined using the Inbody 3.0 (Cheonan, Republic of Korea) based on the BIA method in Ansan/Ansung cohort. The prediction models for body fat and skeletal muscle mass were developed and validated in Ansan/Ansung cohort using a machine learning prediction model [12]. The prediction models generated from the XGBoost and deep neural network (DNN) algorithms were applied to the city-hospital-based cohort, and body fat and skeletal muscle masses were estimated in the participants of a city-hospital-based cohort. The prediction models by XGBoost and DNN showed good accuracy for body fat mass and skeletal muscle (R^2^ = 0.82 and 0.89, respectively) [12]. The predicted values of body fat mass were used for the present study.

A doctor measured blood pressure three times via a sphygmomanometer under resting conditions, and the average systolic blood pressure (SBP) and diastolic blood pressure (DBP) were reported. After fasting for more than 12 h, blood was collected in heparin-treated and non-treated tubes, and the plasma glucose concentrations were measured using a Hitachi 7600 Automatic Analyzer (Hitachi, Tokyo, Japan). HbA1c in heparin-treated blood was measured using an automatic analyzer (ZEUS 9.9; Takeda, Tokyo, Japan). Serum total cholesterol, high-density lipoprotein cholesterol (HDL-C), triglyceride and creatinine concentrations, and alanine aminotransferase (ALT) and aspartate aminotransferase (AST) activities were assessed using a Hitachi 7600 Automatic Analyzer. The serum high-sensitive C-reactive protein (hs-CRP) concentrations were measured using a high-sensitivity ELISA kit (Thermofisher, Waltham, MA, USA). The white blood cells (WBCs) were counted from the EDTA-treated blood. The estimated glomerular filtration rate (eGFR) was calculated using the equation by Modification of the Diet in Renal Disease (MDRD) study [13].

### 2.3. Definition of Obesity and Metabolic Syndrome

Obesity was defined according to total body fat mass estimated by BIA: men with ≥25% (*n* = 3298) and women with ≥30% (*n* = 7204) were considered to be obese and assigned to the high-body-fat (high-BF) group (the case group) by Korean definitions of obesity. Metabolic syndrome is a cluster of energy, glucose, and lipid disorders categorized according to the 2005 revised National Cholesterol Education Program—Adult Treatment Panel III criteria for Asia [14,15]. The criteria for MetS were as follows: (1) abdominal obesity (waist circumference ≥ 90 cm for men and ≥85 cm for women), (2) elevated fasting blood glucose level (≥100 mmol/L) or current use of anti-diabetic medication, (3) elevated blood pressure (average systolic blood pressure ≥ 130 mmHg or diastolic blood pressure ≥ 85 mmHg) or current blood pressure medication use, (4) low HDL-C level (<40 mg/dL for men and <50 mg/dL for women), or (5) elevated serum triglyceride level (≥150 mmol/L) or current use of anti-dyslipidemic medication. Participants meeting three or more criteria were considered to have MetS.

### 2.4. Usual Food Intake Using a Semi-Quantitative Food Frequency Questionnaire (SQFFQ)

During the last 12 months of the interview, the food intake of each participant was determined using an SQFFQ designed for the Korean diet. The SQFFQ accuracy and reproducibility were validated with the three-day food records in four seasons in Koreans [16,17]. The SQFFQ includes 106 food items that Koreans commonly consume, and the food frequencies were categorized into never or seldom, once per month, two to three times monthly, once or twice weekly, three or four times weekly, five or six times weekly, daily, twice daily, and ≥3 times daily. The food amount at a meal was scored as more than, equal to, or less than the regular portion size visualized by photographs of 106 foods. The participants checked the frequencies and the portion size of 106 food items in the SQFFQ. The daily food intake was calculated by multiplying the median of the weekly consumed frequencies by portion sizes. The food intake was given in grams/day. The daily energy, carbohydrates, fat, protein, vitamin, and mineral intakes were calculated from the SQFFQ results using Can-Pro 2.0 nutrient assessment software designed by the Korean Nutrition Society.

### 2.5. Dietary Patterns by Principal Components Analysis

For dietary pattern analysis, 106 food items in the SQFFQ were classified into 30 predefined food groups, as reported previously [18]. The dietary patterns were designed using principal component analysis of the 30 food groups based on eigenvalues > 1.5, and four dietary patterns explained the criteria [18]. The orthogonal rotation procedure (varimax) yielded four dietary patterns uncorrelated with each other, and foods with ≥0.40 factor-loading values were considered the predominant contributors to the assigned dietary pattern [18]. Table 1A lists the predominant foods found as >0.4 of factor-loading values in each dietary pattern. These patterns indicate the participant’s diet types, and they were divided into the KBD, PBD, WSD, or RMD. Nutrient intake in each dietary pattern is shown in Table 1B. Energy intake with high carbohydrates and low fat was lower in RMD, but energy intake with high fat and low carbohydrate was higher in the WSD group.

### 2.6. Dietary Inflammatory Index (DII)

DII, an index of the pro-inflammatory potential of diets, was calculated from the equation with assigned food and nutrient intakes using their dietary inflammatory weights including energy, 32 nutrients, four food products, four spices, and caffeine, as described previously [19]. Because the SQFFQ did not include garlic, ginger, saffron, and turmeric, their intakes were excluded from the DII calculation. DII was calculated by multiplying the dietary inflammatory scores of the 38 food and nutrient components by the daily intakes, and the sums of the scores of 38 items were divided by 100 [19,20].

### 2.7. Genotyping Using a Korean CHIP and Quality Control

The Center for Genome Science at the Korea National Institute of Health determined the participants’ genotypes in the Ansan/Ansung and city-hospital-based cohorts. The genomic DNA was isolated from whole blood, and genotypes were measured using a Korean Chip (Affymetrix, Santa Clara, CA, USA) designed to examine the disease-related single-nucleotide polymorphisms (SNPs) in Koreans [10]. The genotyping accuracy was estimated using Bayesian robust linear modeling in the Mahalanobis distance genotyping algorithm. The inclusion criteria of the genotyping accuracy, missing genotype call rate, and heterozygosity were ≥98%, <4%, and <30%, respectively, and the data showed no gender bias. The genetic variants that were included satisfied the Hardy–Weinberg equilibrium (HWE) at *p* > 0.05 and minor allele frequency (MAF) at >1% [21]. Manhattan and quantile–quantile (Q-Q) plots indicated the accuracy of GWAS data using the Fastman library in the R program [21]. A Manhattan plot of genetic variants was displayed with the negative logarithms of the association of *p*-values for high body fat mass. A Q-Q plot is a probability plot to show the goodness of fit of the actual data distribution to the theoretical data distribution. The Q-Q plot of genotype data displayed the quantile distribution of observed *p*-values (on the *y*-axis) versus the quantile distribution of expected *p*-values (on the *x*-axis). The Q-Q plot was constructed to ensure that the lambda value of the Q-Q plot was close to 1 and confirmed that the GWAS genotypes were ideal. The pathways linked to the genetic variants associated with high body fat mass having *p*-values < 0.05 for Bonferroni correction were selected using the MAGMA gene-set analysis in SNP2GENE of FUMA web application, available through the git repository (https://github.com/Kyoko-wtnb/FUMA-webapp/, accessed on 18 May 2022).

### 2.8. Selection of the Genetic Variants That Influence Obesity Defined by Fat Mass and the Best Model with SNP–SNP Interactions

**Figure 1:**
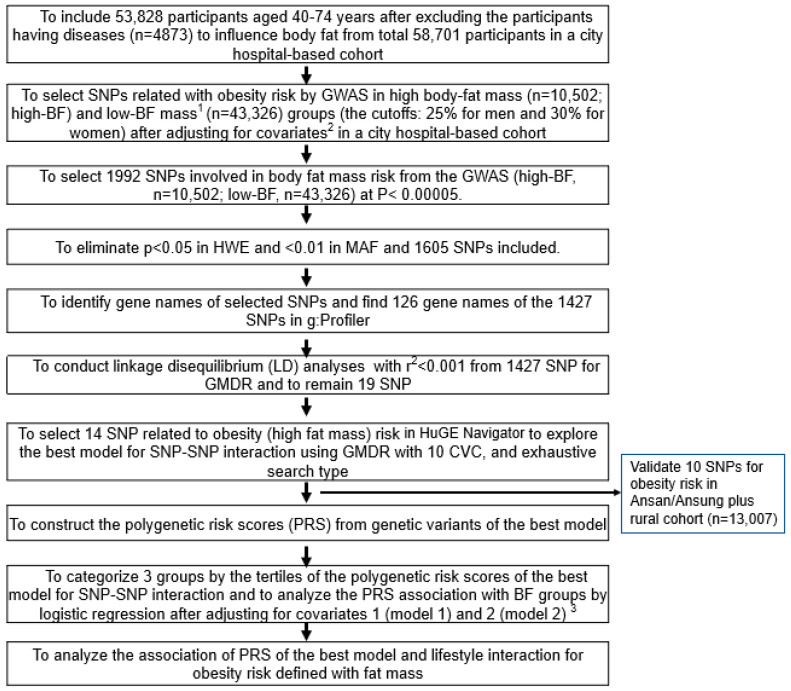
The scheme of searching the genetic variants related to body fat mass and polygenic risk factor (PRS) of the selected genetic variants’ interaction with lifestyles. ^1^ High body fat mass (obesity) was defined as body fat higher than 25% and 30% of body weight for men and women, respectively. ^2^ Covariates included age, gender, residence area, survey year, daily energy intake, and education and income levels. ^3^ Covariates for model 1 were age, gender, residence area, survey year, education, and income; those for model 2 were covariates for model 1 plus energy intake, alcohol intake, regular exercise, and smoking status.

Figure 1 presents the procedure of selecting genetic variants for high body fat risk and investigating the best model for SNP–SNP interactions. The GWAS was conducted to explore genetic variants associated with obesity risk in the urban-hospital-based cohort (*p* < 5 × 10^−5^). From the GWAS associated with obesity risk, 1992 genetic variants were selected at *p* < 5 × 10^−5^. We eliminated 387 genetic variants that did not meet MAF (<1%) and HWE (*p* < 0.05). In the gene name search using g:Profiler (https://biit.cs.ut.ee/gprofiler/snpense, accessed on 2 June 2022), 178 SNPs were not identified with gene names, and 126 gene names of the 1427 genetic variants were identified. The linkage disequilibrium (LD) analyses were performed on the SNPs of the 1427 genetic variants using Haploview 4.2 in PLINK. The potential genetic variants in the same chromosome were not strongly correlated (D’ < 0.2). The SNPs with high D’ values were not included in the generalized multifactor dimensionality reduction (GMDR) because they provided the same information on the genetic impact. There were 19 SNPs in 18 genes selected, and genes associated with fat mass were selected using HuGE Navigator (https://phgkb.cdc.gov/PHGKB/hNHome.action, accessed on 20 June 2022).

Among the 14 genetic variants selected for obesity risk, ten SNPs with an SNP–SNP interaction were selected automatically by GMDR. The best SNP–SNP interaction model was selected in a sign-rank test of trained balanced accuracy (TRBA) and testing balanced accuracy (TEBA) while adjusting for the covariates using a GMDR program and a *p*-value threshold of 0.05 [10]. The covariates used were age, gender, survey year, residence area, education, and income for models 1 and 2 plus energy intake, alcohol intake, regular exercise, and smoking status when carried out GMDR and logistic regression between BF groups and genetic variants. Models 1 and 2 indicated the results with different covariates. Ten-fold cross-validation was also checked for cross-validation consistency (CVC) because the sample size was larger than 1000 [22]. In total, 10 out of 10 in the CVC met the perfect cross-validation criteria. The association between body fat mass and ten SNPs selected from a city-hospital-based cohort was validated in the Ansan/Ansung plus rural cohorts (*n* = 13,007). Their significance level was considered as 0.05 since the validation was conducted in the selected genetic variants.

The risk allele number of each SNP was counted to generate the PRS of the best model. For example, the genetic score for the SNP was 2, 1, and 0 when the participants had AA, AG, and GG of one SNP, and the A allele was the risk allele, respectively. The polygenic risk score (PRS) of the best model was assessed by summing the number of the risk alleles from each selected SNP in the best gene–gene interaction model [3,23]. The criteria of the best mode were the *p*-value of the sign test for TEBA, which was 0.001, and CVC, which was 10. The two models with the smallest genetic variants were selected among the models meeting the criteria. From the GMDR analysis, the PRS in the three- and six-SNP models were divided into three categories according to the number of risk alleles. They were classified as low PRS, middle PRS, and high PRS when the number of risk alleles in the PRS was 0–2 (*n* = 19,686), 3–4 (*n* = 30,513), and ≥5 (*n* = 3629) in the three-SNP model and 0–5 (*n* = 27,212), 6–7 (*n* = 20375), and ≥8 (*n* = 1822) in the six-SNP model, respectively. Among the best models to meet the *p*-value of the sign test and CVC, the model with the lowest SNP number (three-SNP model) was used to interact with the lifestyle parameters.

### 2.9. Expression Quantitative Trait Locus (eQTL) Analysis

The eQTL analysis is a direct approach to estimating the candidate gene expression of the genetic variants at risk loci. The allele variants are involved in the corresponding gene expression, and the expression of candidate susceptible genes with risk alleles is estimated to influence various diseases [23]. Gene expressions corresponding to the genetic variants related to the abdominal obesity risk were identified by eQTL analysis in the Genotype-Tissue Expression (GTE) × eQTL calculator (https://gtexportal.org/home/testyourown, accessed on 19 July 2021). The gene expressions in the subcutaneous and visceral adipose tissues, skeletal muscles, liver, and brain were calculated using the GTE × eQTL calculator.

### 2.10. Statistical Analysis

The statistical analysis was performed using SAS (version 9.3; SAS Institute, Cary, NC, USA). A sample size of 53,828 was sufficient to achieve significance at α = 0.05 and β = 0.99 at an odds ratio of 1.05 in the logistic analysis using a G-power calculator. The descriptive statistics for categorical variables, such as gender and dietary habits, were obtained by determining the frequency distributions, which were analyzed statistically according to the low-BF and high-BF groups of the classification variables using a chi-square test. Descriptive statistics of the continuous variables were analyzed as the adjusted means with standard deviations after adjusting for the covariates. The statistical differences among the gender and insulin-resistance groups were compared using a two-way analysis of covariance (ANCOVA) [24]. Multiple comparisons of the groups were performed using Tukey’s test.

The association of insulin resistance on metabolic parameters was examined by logistic regression analysis with the low-BF group as the reference after adjustment for covariates. The results are presented as the odds ratios (ORs) and 95% confidence intervals (CI) of each biochemical parameter for the high-BF and low-BF groups. The first model was generated after adjusting for age, residence area, survey year, lean body mass, education, and income. The second model was produced with the adjustments for covariates in model 1 and the energy intake, physical activity, smoking status, and alcohol consumption.

The lifestyle-related parameters were categorized into the high or low groups using the criteria defined by the predesignated cutoffs, such as the dietary reference intake or 30th percentiles of each variable, to determine the interactions between the fat mass and lifestyle parameters. Two-way ANCOVA was used to analyze the interactions between the fat mass groups and the lifestyle parameters, including dietary intake, smoking, and physical activity. The main effects were insulin resistance and lifestyle-related parameters with their interaction terms after adjusting for covariates. The ORs and 95% CI of fat mass with lifestyle-related parameters were also calculated by logistic regression analysis in the high-BF and low-BF groups of the lifestyle-related parameters. The significant difference in the high-BF percentage was analyzed using the PRS groups in the χ^2^ test in the low and high groups of lifestyle-related parameters.

## 3. Results

### 3.1. Demographic Characteristics and Lifestyles According to Genders and Obesity

Age was significantly higher in the high-BF group than in the low-BF group for both genders. Education and income also significantly affected body fat mass for both genders: The participants with less than a high school education had a significantly higher incidence of high body fat for both genders. Men in the lowest income group had a significantly lower incidence of high body fat, whereas women in the lowest income group had a significantly higher incidence of high body fat mass (Table 2). Energy intake was not significantly different between the high- and low-BF groups in women; however, men in the low-BF group had slightly but significantly higher energy intakes than those in the high-BF group. Carbohydrate and fat proportions were not significantly different according to body fat. In dietary fat composition, MUFA intake was higher, and PUFA intake was lower in the high-BF group than in the low-BF group. Vitamin C, fiber, and Ca intakes were lower in the high-BF group than in the low-BF group only in women, but vitamin D intake showed the same trend in both genders (Table 2). DII was the same between fat mass groups in both genders although DII was higher in men than women. Among dietary patterns, the number of men consuming a KBD was higher in the high-fat mass group, but it was the opposite in women. The number of participants with high PBD was lower in the high-BF group only in women, but those with a high WSD were higher in the high-BF group only in men (Table 2). The participants who were smokers were lower in number in the high-BF group than the low-BF group only in men. Alcohol intake did not differ between low- and high-BF groups for either gender. However, the number of participants who participated in regular exercise was lower in the high-BF group than in the low-BF group (Table 2).

### 3.2. Anthropometric and Biochemical Parameters According to Genders and Obesity

The participants with high body fat mass were shorter and had higher BMIs and waist circumferences but lower SMI than those with low body fat mass (Table 3). Height and SMI were inversely associated with body fat mass, and BMI and waist circumferences were positively linked to body fat mass. MetS was higher in the high-BF group than in the low-BF group and positively associated with body fat mass by 6.29 times (Table 3). Serum glucose and blood HbA1c concentrations were significantly lower in men in the low-BF group than in men in the high-BF group. Serum glucose but not HbA1c was significantly lower in women in the low-BF group as compared to the high-BF group (Table 3). Furthermore, dyslipidemia, including hypercholesterolemia, hyper-LDL-cholesterolemia, hypo-HDL-cholesterolemia, and hypertriglyceridemia, was also positively linked to fat mass (Table 3). However, hypertension was less associated with fat mass. eGFR was lower in high-BF than low-BF in men but not women. Serum AST and ALT concentrations, known as liver damage indexes, were higher in the high-BF group than in the low-BF group, whereas they were positively associated with fat mass (Table 3). Serum hs-CRP concentrations were higher in the high-BF group than in the low-BF group and were positively related to fat mass (Table 3).

### 3.3. Polygenetic Variants and Their Interactions Related to Obesity Defined by Fat Mass

**Figure 2:**
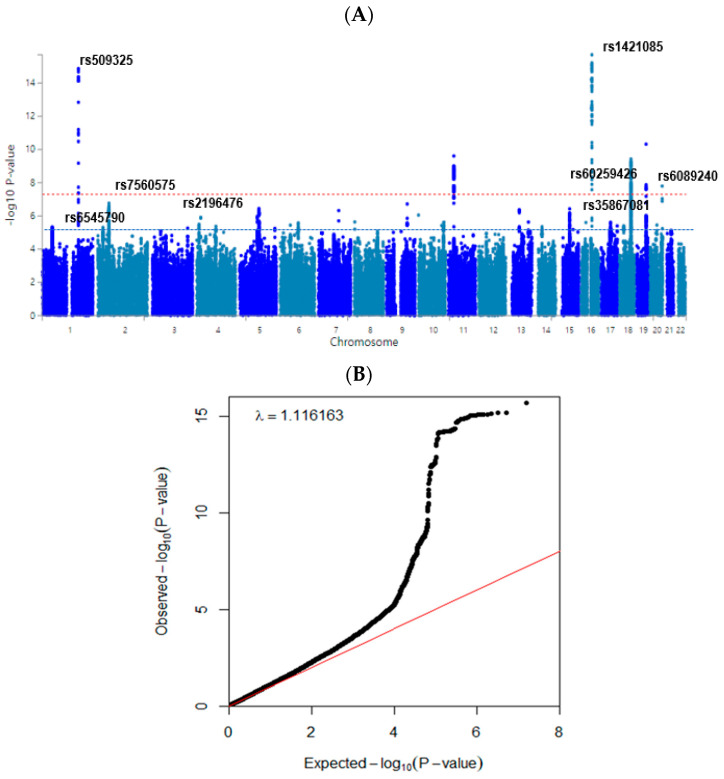
Distribution of genetic variants for high body fat mass risk by genome-wide association study. (**A**) Manhattan plot of the *p*-value of genetic variants for high body fat risk. Red-dot and blue-dot lines indicate the *p*-value of 5 × 10^−8^ and 5 × 10^−5^. (**B**) Q–Q plot of observed and expected *p*-values for body fat risk. Red and black lines represent theoretical and actual distributions, respectively.

The statistical association of genetic variants with body fat mass is shown in a Manhattan plot (Figure 2A). The Q-Q plot (Figure 2B) shows the quantile distribution of the log of observed *p*-values versus that of the log of expected *p*-values. The lambda value was 1.116. These results suggested that genetic variants for fat mass were in an acceptable range although they might be somewhat inflated.

The gene-set analyses used the full distribution of SNP *p*-values, and we found which GO pathways were related to the prioritized genes. The genes of selected genetic variants found in GWAS between low-BF and high-BF groups were clustered with the GO pathways. Table 4 shows the GO pathways related to the genetic variants linked to body fat mass (*p* < 0.0002). Genes of the genetic variants associated with fat mass risk were highly involved in the sodium ion transmembrane transport pathway in the biological process of gene ontology (GO) (Table 4). Interestingly, the genes were associated with myoblast proliferation and the apoptotic process of neurons in the biological process of GO. In the curated gene sets, the biocarta flumazenil pathway and Reactome signaling to p38 via RIT and RIN were associated with genes related to fat mass (Table 4). These pathways were related to toxic compound metabolism by MAPK and RAS.

Ten genetic variants with the genetic variant–genetic variant interaction are presented in Table 5. The ten genetic variants were rs509325_*SEC16B*, rs6545790_*ADCY3*, rs7560575_*PSME4*, rs2196476_*SLIT2*, rs6265_*BDNF*, rs587056_*FARP1*, rs1421085_*FTO*, rs35867081_*BAIAP2*, rs60259426_*SYMPK*, and rs6089240_*CDH4*. The genes are known to be involved in obesity. They met the MAF > 1% and *p*-value for HWE > 0.05, whereas the adjusted ORs were 0.9278–1.35, and their *p*-values were <5 × 10^−6^ and <0.0069 in the city-hospital-based cohort and Ansan/Ansung cohort plus rural cohort, respectively.

### 3.4. The Gene Expression According to the Alleles of the Selected SNPs in Different Tissues from GTEx v8

**Figure 3:**
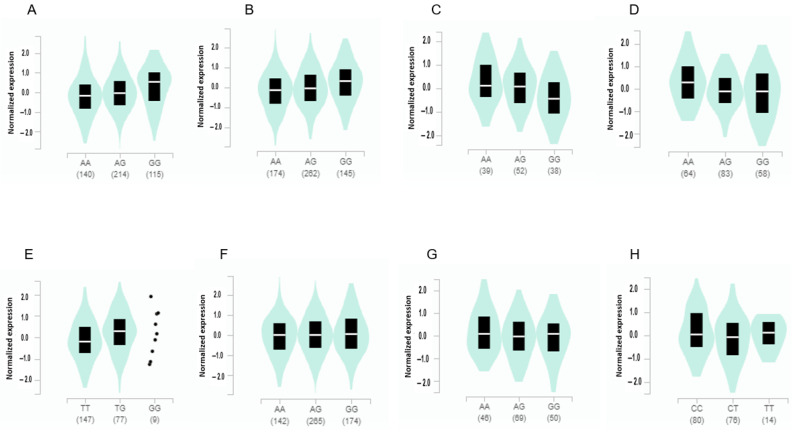
Gene expression according to the alleles of the selected SNPs for high body fat risk in different tissues. (**A**) *ADCY3*_rs6545790 in visceral adipose tissues (slope of the SNP alleles = 0.21, *p* = 4.1 × 10^−14^). (**B**) *ADCY3*_rs6545790 in subcutaneous adipose tissues (slope of the SNP alleles = 0.23, *p* = 2.2 × 10^−19^). (**C**) *ADCY3*_rs6545790 in the amygdala (slope of the SNP alleles = −0.18, *p* = 0.0064). (**D**) *ADCY3*_rs6545790 in the brain cortex (slope of the SNP alleles = −0.28, *p* = 5.4 × 10^−10^). (**E**) *SEC16B*_rs509325 in the adrenal gland (slope of the SNP alleles = 0.24, *p* = 0.0026). (**F**) *BAIAP2*_rs35867081 in subcutaneous adipose tissues (slope of the SNP alleles = 0.066, *p* = 0.025). (**G**) *BAIAP2*_rs35867081 in the hippocampus (slope of the SNP alleles = 0.066, *p* = 0.025). (**H**) *FARP1*_rs587056 in the hypothalamus (slope of the SNP alleles = −0.19, *p* = 0.00072).

The expression of *ADCY3* with the minor allele of rs6545790 was elevated with 0.21 of slope in visceral fat (*p* = 4.1 × 10^−14^) and 0.23 in subcutaneous fat (*p* = 2.2 × 10^−19^) (Figure 3). It indicated that *ADCY3* having a risk allele had an increased expression compared to that having a non-risk allele. By contrast, it significantly decreased with a −0.18 and −0.28 slope in the amygdala (*p* = 0.0064) and brain cortex (*p* = 5.4 × 10^−10^), respectively. The *SEC16B* expression with the minor allele of rs509325 increased with a slope of 0.24 in the adrenal gland. The *BAIAP2* expression with the minor allele of rs35867081 was elevated with a 0.066 (*p* = 0.025) and −0.14 (*p* = 0.000035) slope in the subcutaneous adipose tissue and the hippocampus, respectively (Figure 3). However, the minor allele of *BDNF*_rs6265 was not significantly associated with gene expression (*p* = 0.55) although it was a missense mutation.

### 3.5. The Best Model of Genetic Variants with SNP–SNP Interaction for Obesity

**Figure 4:**
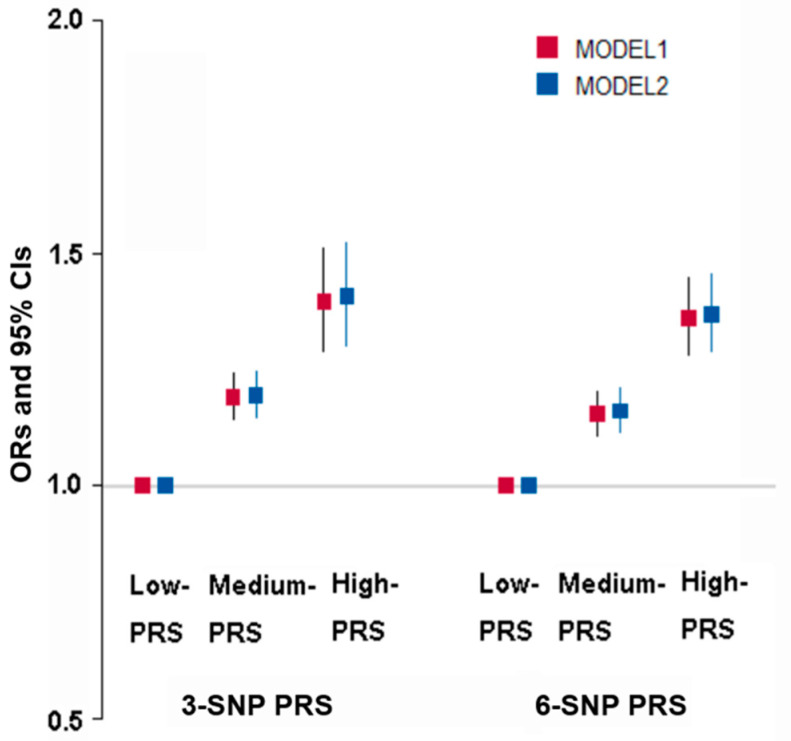
Adjusted odds ratio (ORs) and 95% confidence intervals (CI) of three-SNP PRS and six-SNP PRS for obesity defined with body fat mass. PRS was generated with the sum of the number of risk alleles in each SNP, and it was classified as low PRS, medium PRS, and high PRS according to the PRS was 0–2, 3–4, and ≥5 in the three-SNP model and 0–5, 6–7, and ≥8 in the six-SNP model, respectively. Models 1 and 2 were conducted with different covariates. Covariates were age, gender, residence area, education, and income for models 1 and 2 plus energy intake, alcohol intake, regular exercise, and smoking status.

The best models with genetic variant–genetic variant interactions influencing obesity determined by fat mass were selected when satisfying *p*-value < 0.05 for the sign test of TEBA and CVC 10/10. The models to meet the criteria included 3-, 6-, 7-, 8-, and 10-SNPs (Supplementary Table S1). The three-SNP model included *BDNF*_rs6265, *FTO*_rs1421085, and *SEC16B*_rs509325. In the three-SNP model, the PRS was associated with body fat mass by 1.408 and 1.396 times after adjusting for covariates 1 and 2, respectively (Figure 4). The six-SNP model contained *ADCY3*_rs6545790, *BDNF*_rs6265, *SEC16B*_rs509325, *BAIAP2*_rs35867081, *SYMPK*_rs60259426, and *CDH4*_rs6089240. The PRS of the six-SNP model was related to body fat mass by 1.36 and 1.37 times, respectively. However, the adjusted ORs in the six-SNP model were decreased compared to the three-SNP model (Figure 4). Therefore, the three-SNP model was used as the best model for body fat mass risk.

### 3.6. Interaction of PRS with Lifestyles to Influence Obesity

**Figure 5:**
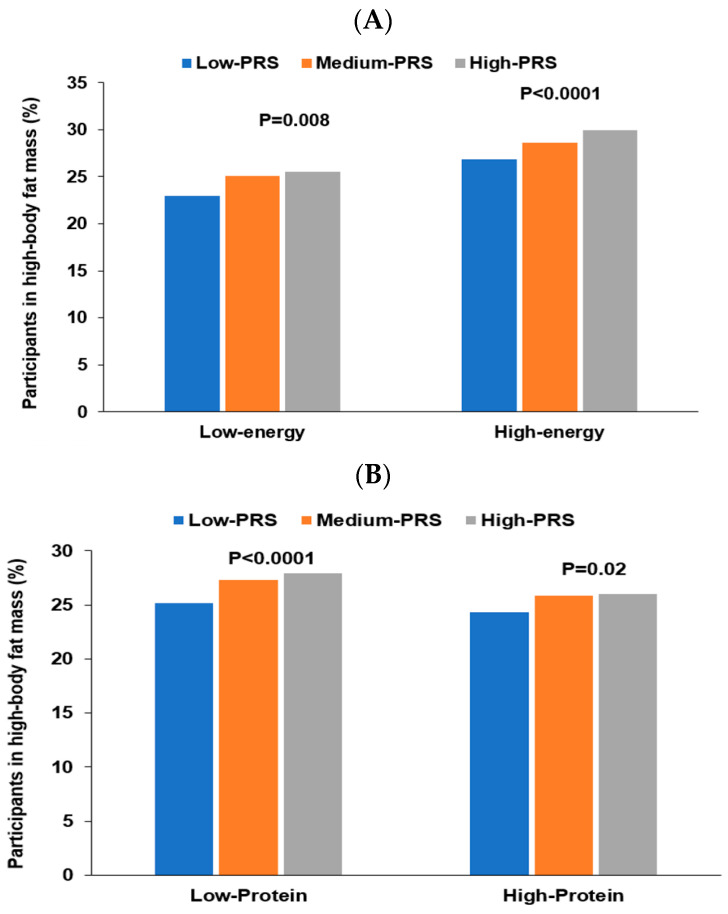

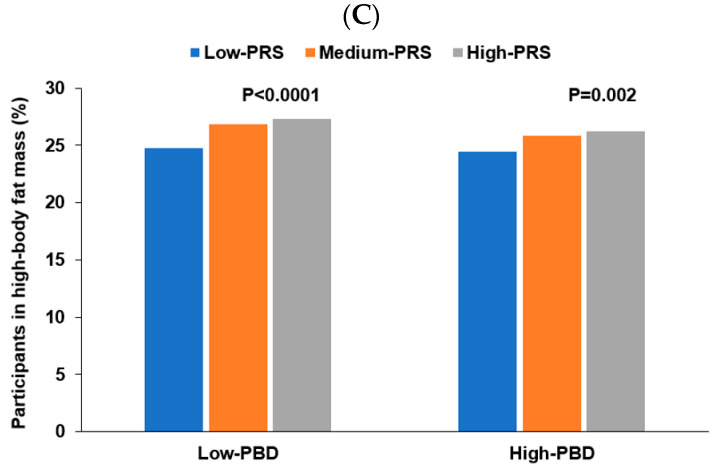
Percentage of the participants with high body fat mass. (**A**) According to energy intake. (**B**) According to protein intake. (**C**) According to a plant-based diet.

Energy intake interacted with PRS of the three-SNP model to affect body fat mass, and positive associations of PRS with body fat mass risk were 1.228 and 1.157 times, respectively, in low and high energy intakes (Table 6). The proportion of the participants was higher in the high-PRS group than in the low-PRS group in both low and high energy intake (Figure 5A). The results suggested that the participants with high PRS increased fat mass even in low energy intake. Protein intake also interacted with PRS to influence fat mass (Table 6). A high protein intake could prevent the increase of fat mass in the participants in the high-PRS group compared to those in the low-PRS group (Figure 5B). Other nutrient intakes did not interact with PRS affecting fat mass. Among four dietary patterns, only PBD intake interacted with PRS for fat mass (Table 6). The high body fat mass proportion was higher in the participants with high PRS than those with low PRS in both low and high PBD intake. However, the increase was much more significant in the low PBD intake than in the high PRB intake (Figure 5C). Exercise and smoking status did not interact with PRS to affect fat mass. Therefore, these results suggest that Koreans with high PRS can decrease their risk of high body fat by consuming a PBD diet with less energy and high protein intake.

## 4. Discussion

The present study investigated genetic, lifestyle, and dietary factors that differentiate people with healthy fat mass levels from those with excessive body fat. The study utilized a genome-wide association study (GWAS) followed by an analysis of PRS for high body fat mass (obesity) and comparisons of body fat mass status in subjects according to high and low risk scores. This approach was used to highlight what dietary and lifestyle patterns might lead to an individual being lean despite a high genetic risk of obesity and vice versa.

This study also looked at four dietary patterns: KBD, PBD, WSD, and RMD. The diets varied in energy and nutrient content, varying from 179% of the estimated energy requirements for the WSD to 87% for the RMD. Nevertheless, there was no clear distinction between the dietary patterns except that only the PBD was clearly associated with a lower fat mass (110% of the estimated energy requirements). This may not be too surprising. In a recent perspective article, Mozaffarian published a graph that clearly showed a large increase in the percentage of obesity in the USA population from the year 2000 (30%) to 2018 (42%) [25]. Despite the rapid increase in the obesity rate, there was no change in energy intake. It highlights the need to look for causes of obesity beyond energy intake. This study attempts to contribute to understanding the dietary causes of obesity while realizing that there are unlikely to be straightforward explanations.

The three genetic variants constituted the best model for high body fat mass contained *BDNF*, *FTO*, and *SEC16B* in the present study. Genetic variants that result in decreased production of BDNF have been shown to increase the obesity risk significantly in humans [26]. The *BDNF*_rs6265 variant causes a methionine substitution for valine, which results in multiple physiological and psychological changes, including impaired fat oxidation in muscle, type 2 diabetes, depression, and disruption of hunger and satiation signals [24,27]. Interestingly, the consequences of the *BDNF*_rs6265 are associated with obesity risk, and it interacts with lifestyles, including nutrient intake [28]. Furthermore, it interacts with other obesity-related genetic variants to exacerbate obesity risk, and PRS, including *BDNF*_rs6265, interacts with lifestyles to modulate the obesity risk [3]. These results suggest that neuronal circuit integrity, survival, and synaptic plasticity may be associated with obesity risk.

The *FTO* gene, located at chromosome 16q12.2, encodes for the fat-mass- and obesity-associated protein. The *FTO* protein functions as a nucleic acid demethylase, and thus, it is involved in controlling the methylation of DNA and RNA. However, the *FTO*_rs1421085 non-coding variant does not affect the expression or the functionality of the FTO protein. Mutated *FTO* (rs1421085) interacts with Iroquois-class homeodomain proteins IRX-3 and IRX-5, which are also linked to obesity in humans and animals [28,29]. Laber et al. [30] have demonstrated that the *FTO*_rs 1421085 interaction with IRS-3 and IRX-5 modulates numerous organismal phenotypes related to diet-induced weight gain and observes that the *FTO*_rs1421085 genetic variant mediates cross-species conserved changes in steroid patterns following nutritional challenges.

The third member of the best model group, *SEC16B*_rs509325, is a variant of the *SEC16B* gene, also known as *RGPR*-p117, a gene that encodes a protein known as regucalcin that is characterized by its leucine zipper motif. Regucalcin is known to be a transporter of proteins from the endoplasmic reticulum, but evidence to date has not demonstrated the role of the transporter in lipid metabolism [31]. Nevertheless, variants of the *SEC16B* gene have consistently been shown to be linked to human obesity, especially in Asian populations [3,32]. Although there has not been any link to fat metabolism established for *SEC16B*, it is linked to transport peptides involved in regulating satiety, including neuropeptide Y and proopiomelanocortin [3,33]. All three genes composing the best model affect different aspects of metabolism.

One of the objectives of this study was to determine which dietary patterns and/or dietary factors might help overcome a genetic propensity to obesity. Three factors interacted with the PRS to lower obesity risk. A plant-based diet was the most highly significant factor (*p* = 0.0026) in ameliorating the risk of obesity in participants with a high genetic risk for obesity. KBD, WSD, and RBD had no statistically significant interaction with a high PRS. Energy intake and protein intake also significantly interacted with a high PRS, and a high-protein diet seemed to mostly eradicate the effect of a high PRS on fat mass.

The most important finding of the current study was that the primary dietary factors linked to low body fat content other than lower energy intake were high dietary protein consumption and, even more potent, a more plant-based diet. Among the four dietary patterns studied, most had no significant effect on the risk for high body fat content; however, diets considered low in plant-based foods and low in protein resulted in a significantly higher percentage of obese subjects among those with medium and high PRS. This result seems counterintuitive since plant-based diets are typically lower in protein than diets high in animal foods [34]. However, these results concur with studies in other ethnic groups that have found that vegetarian and/or plant-based diets support a leaner body type [35,36].

There are various hypotheses about how a plant-based diet may help maintain a healthy body weight. First, a plant-based diet has a lower energy density, resulting in greater satiety and lower energy consumption [22,36]. Furthermore, plant-based diets contain lower fat and higher fiber, which may impact the microbiome, which is known to have substantive impacts on energy utilization in humans [37,38,39]. However, microbiome research has not progressed to the point that changes in the gut microbiome can be considered a cause or cure for excess body weight. There is also evidence that various phytochemicals from plant-based foods and dietary supplements may have anti-obesity effects by various mechanisms [23]. It has been shown that the anti-inflammatory properties of plant-based diets can prevent obesity and even result in a healthier obese phenotype in people who become obese [40]. The present study revealed a weak inverse association between high body fat mass and dietary inflammation index (OR = 0.927; 95% CI = 0884–0.973) despite a high inverse association between PBD and DII (OR = 0.368; 95% CI = 0.348–0.339). Even though it has been difficult to demonstrate specific phytochemicals that profoundly affect body fat mass, it has been previously demonstrated that a phytochemical index score was associated with a lower prevalence of obesity in the Korean population [41]. Therefore, the accumulation of current evidence supports the finding in this study that consuming a plant-based diet provides significant benefits for managing body fat.

Previous studies have also found an association between higher protein intake and lower body fat mass. It has also been observed in a recent meta-analysis and systematic review that diets high in protein (18–59 energy %) were associated with an average of 1.6 kg decrease in body weight [42]. The present study did not determine a more precise dietary protein level needed for weight reduction. However, the efficacy of increased protein intake for weight loss has support from a human trial with biscuits enriched with plant-based protein. The cutoff of protein intake was 15 En%, so our results suggest that higher than 15 En% from protein may decrease fat mass. The previous study found that the group with the protein-added biscuits lost significantly more body weight and fat mass than the control group [43].

In this study, having a plant-based diet had the most significant association with lower body fat, followed by a high-protein diet. It suggests that a plant-based diet high in protein might be optimal for the Korean population and possibly people of other ethnic groups. The findings support the possibility that substituting plant protein for animal protein can decrease the incidence of type 2 diabetes, probably by decreasing obesity-induced inflammation [44]. It was also demonstrated that elderly Korean men with concurrent sarcopenia, osteoporosis, and obesity had the same total and animal protein intake but a significantly lower intake of plant protein than controls [45].

This research revealed that modest differences in dietary choices could have a significant impact on fat accumulation in the Korean population. Other studies have shown that people who adhere to vegetarian diets are less obese than their omnivorous counterparts [46] and that vegetarians are healthier even at the same BMI [47]. However, the subjects in the present study with a plant-based diet were not necessarily vegetarians. A plant-based diet in this study indicated that plant foods contributed relatively more to the total energy intake than animal foods. However, the subjects did not adhere to any particular dietary restrictions. The lack of a clearly defined diet could be both a strength and a limitation of the study. There was more variability in the dietary type and potentially less of an effect than if all subjects with a plant-based diet were vegetarians. On the other hand, this represents a more “real world” scenario for many people and shows that modest changes can have profound effects.

## 5. Conclusions

In conclusion, this study suggested that *BDNF*_rs6265, *FTO*_rs1421085, and *SEC16B*_rs509325 interact with high body fat mass, that plant-based and high-protein diets are beneficial for maintaining a lower body fat in adults with a high PRS of the three genetic variants. The *ADCY3*_rs6545790 and *BAIAP2*_rs35867081 minor alleles included in the three-SNP model increased their gene expression in the visceral and subcutaneous adipocytes. However, their gene expression decreased in the hypothalamus in eQTL analysis, suggesting the changes in their expression could be involved in increased body fat mass. This study also demonstrated that the discovery of PRS–environment interactions in body fat was applicable in the clinical setting. Therapies targeting the PRS could help prevent obesity and manage weight by applying personalized nutrition programs emphasizing a plant-based diet high in protein. A large clinical randomized trial is needed to show the cause-and-effect relationship between the three SNP combinations and PBD in the development of obesity. If confirmed, it can be applied to personalized nutrition.

## Figures and Tables

**Table 1:** (**A**) The factor-loading values of the predefined 29 food groups in each dietary pattern. (**B**) Energy intake in each dietary pattern.

(**A**)								
	**Korean Balanced Diet**		**Plant-Based Diet**		**Western-Style Diet**		**Rice-Main Diet**	
Rice	−3		−7		6		93	*
Whole grain	8		−4		−3		−93	*
Bread	−1		5		63	*	1	
Cookie	−8		37		50		−5	
Noodles	−8		33		29	*	6	
Bean	32		47	*	3		2	
Potato	26		49	*	4		−3	
Kimchi	50	*	0		0		−2	
Egg	8		44	*	15		4	
Fast food	−5		18		73	*	−4	
Green vegetables	68	*	40	*	−2		−1	
White vegetables	71	*	27		3		2	
Mushroom	50	*	34		−6		−3	
Fatty fish	53	*	21		12		0	
Whitefish	66	*	15		15		0	
Crab	48	*	3		21		1	
Processed meats	18		15		7		−1	
Red meat	43	*	−6		44	*	7	
Chicken	16		4		63		−5	
Soups	32		−5		40	*	3	
Seaweeds	45	*	39	*	−2		−4	
Milk	12		49	*	1		0	
Beverage	20		31		6		2	
Coffee	9		0		20		14	
Tea	12		−7		26		13	
Fruits	21		46	*	−6		−5	
Pickles	50	*	−2		6		2	
Alcohol	16		−27		18		5	
Nuts	−1		50	*	5		−5	
Variance explained by each dietary pattern	3.57		2.44		2.30		1.79	
(**B**)								
	KBD		PBD		WSD		RMD	
Energy intake (EER%)	125.6 ± 0.434 ^b^		109.8 ± 0.254 ^c^		178.9 ± 1.193 ^a^		87.1 ± 0.143 ^c^	
Carbohydrates (En%)	64.8_0.103 ^c^		69.4 ± 0.061 ^b^		63.7 ± 0.287 ^d^		73.2 ± 0.04 ^a^	
Protein (EN%)	17.1 ± 0.037 ^a^		14.0 ± 0.02 ^b^		14.8 ± 0.103 ^b^		12.8 ± 0.012 ^c^	
Fat (En%)	18.0 ± 0.08 ^b^		16.3 ± 0.047 ^c^		20.8 ± 0.221 ^a^		12.6 ± 0.02 ^d^	
SFA (En%)	4.15 + 0.13 ^d^		5.67 + 0.42 ^b^		4.89 + 0.24 ^c^		0.64 + 0.12 ^a^	
MUFA (En%)	5.18 + 0.15 ^d^		6.89 + 0.45 ^b^		6.13 + 0.257 ^c^		8.87 + 0.12 ^a^	
PUFA (En%)	2.93 + 0.16 ^d^		4.06 + 0.38 ^b^		3.51 + 0.22 ^c^		4.75 + 0.11 ^a^	
Fiber (g)	26.0 ± 0.123		15.1 ± 0.07		14.1 ± 0.34		13.3 ± 0.04	
Vitamin C (mg)	169.7 ± 0.885 ^a^		125.5 ± 0507 ^b^		54.5 ± 2.44 ^d^		91.6 ± 0.289 ^c^	

Values were factor-loading values. When their absolute values were >0.4, they were flagged by *, and the name of each dietary pattern was assigned from the predominant food groups. EER, estimated energy requirement; En%, energy %; SAF, saturated fatty acid; MUFA, monounsaturated fatty acid; PUFA, polyunsaturated fatty acid. ^a,b,c,d^ different letters on the bar indicate significant differences among the groups in Tukey’s test at *p* < 0.05.

**Table 2:** Demographic parameters and nutrient intake according to gender and body fat mass (BF).

	Men (*n* = 19,444)		Women (*n* = 34,384)	
	Low-BF (*n* = 16,146)	High-BF (*n* = 3298)	Low-BF (*n* = 27,180)	High-BF (*n* = 7204)
Age (year)	55.7 ± 0.06 ^b^	56.9 ± 0.13 ^a^	51.9 ± 0.05 ^d^	53.8 ± 0.09 ^c^***^+++###^
Education ≤ Middle school	1318 (13.2)	343 (16.8) ^‡‡‡^	4290 (20.3)	1727 (28.4) ^‡‡‡^
High school	7540 (76.2)	1491 (72.8)	15,479 (73.4)	4074 (67.0)
≥College	1040 (10.5)	214 (10.5)	1322 (6.27)	276 (4.54)
Income				
≤USD 2000	1258 (8.24)	251 (7.76) ‡	2848 (11.2)	868 (12.4) ^‡‡‡^
USD 2000–4000	6565 (42.8)	1311 (40.5)	10,903 (43.0)	3369 (48.0)
>USD 4000	7479 (49.0)	1672 (51.7)	11,579 (45.7)	2777 (39.6)
Energy (EER%)	86.0 ± 0.06 ^b^	85.4 ± 0.13 ^c^	104 ± 0.05 ^a^	104 ± 0.09 ^a^***^+++^
Carbohydrates (En%)	71.6 ± 0.08	71.3 ± 0.17	71.7 ± 0.06	71.6 ± 0.12 ^++^
Proteins (En%)	13.3 ± 0.03 ^b^	13.4 ± 0.05 ^b^	13.6 ± 0.02 ^a^	13.6 ± 0.04 ^a^***^#^
Fat (En%)	13.9 ± 0.06	14.2 ± 0.12	13.9 ± 0.04	14.1 ± 0.09
SFA (En%)	4.46 ± 0.02	4.55 ± 0.06	4.45 ± 0.02	4.43 + 0.04
MUFA (En%)	5.62 ± 0.03 ^a^	5.80 ± 0.06 ^a^	5.45 ± 0.02 ^b^	5.48 ± 0.05 ^b^***^+^
PUFA (En%)	3.26 ± 0.03 ^a^	3.21 ± 0.05 ^a^	3.12 ± 0.02 ^b^	3.07 ± 0.04 ^b^**
Cholesterol (mg/d)	169 ± 1.07	173 ± 2.25	170 ± 0.81	171 ± 1.52
Vitamin C (mg/d)	93.8 ± 0.68 ^c^	91.6 ± 1.50 ^c^	121 ± 0.53 ^a^	115 ± 1.05 ^b^***^++^
Vitamin D (ug/d)	5.68 ± 0.05 ^c^	5.39 ± 0.10 ^d^	6.94 ± 0.04 ^a^	6.57 ± 0.07 ^b^***^+++^
Fiber (g/d)	14.6 ± 0.09 ^b^	14.1 ± 0.20 ^b^	15.3 ± 0.07 ^a^	14.4 ± 0.14 ^b^***^+++^
DII (scores)	−19.96 ± 0.02 ^b^	−21.07 ± 0.04 ^b^	−21.26 ± 0.01 ^a^	−21.34 ± 0.03 ^a^**^+#^
Ca (mg/d)	417 ± 3.42 ^b^	414 ± 5.56 ^b^	491 ± 2.31 ^a^	484 ± 3.81 ^a^***
KBD (Yes, %)	6169 (38.2)	1324(40.2) ^‡^	7379 (31.1)	3057 (28.7) ^‡‡‡^
PBD (Yes, %)	3399 (21.1)	693 (21.0)	9675 (40.7)	4136(38.9) ^‡‡^
WSD (Yes, %)	8218 (50.9)	1877 (50.9) ^‡‡‡^	7871 (33.2)	3684 (34.6) ^‡‡^
RMD (Yes, %)	5089 (31.5)	1016 (30.8)	8180 (34.5)	3628 (34.1)
Non-Smokers (Yes, %)	4712 (29.3)	834 (25.3) ^‡‡‡^	22,864 (96.8)	10,296 (96.9)
Former smokers (Yes, %)	6770 (42.1)	1546 (46.9)	284 (1.2)	132(1.24)
Smokers (Yes, %)	4612 (28.7)	917 (27.8)	532 (1.97)	203 (1.91)
Alcohol drinking (g/day)	35.1 ± 0.40 ^b^	38.9 ± 0.84 ^a^	5.33 ± 0.29 ^c^	5.51 ± 0.56 ^c^***^+++###^
Regular exercise (Yes, %)	10155 (60.5)	1797 (52.4) ^‡‡‡^	16350 (54.0)	3674 (45.9) ^‡‡‡^

Values represent adjusted means ± standard error for continuous variables and the number and percentages of participants for categorical variables. Adjusted for the covariates including age, BMI, education, income, energy intake, alcohol intake, smoking, and total activity. High body fat (case) was defined as 25% and 30% of body weight for men and women, respectively. EER%, the percentage of energy intake based on estimated energy requirement; En%, energy intake percentage. ** Significantly different by genders at *p* < 0.01; *** at *p* < 0.001. + Significantly different by fat mass at *p* < 0.05; ++ at *p* < 0.01; +++ at *p* < 0.001. # Significant interactions at *p* < 0.05; ### at *p* < 0.001. ^‡^ Significantly different from the low-fat mass group at *p* < 0.05; ^‡‡^ at *p* < 0.01; ^‡‡‡^ at *p* < 0.001. ^a,b,c,d^, different letters on the bar indicate significant differences among the groups in Tukey’s test at *p* < 0.05.

**Table 3:** Adjusted means and association of anthropometric and biochemical variables in the urban-hospital-based cohort according to genders and body fat mass (BF).

	Men (*n* = 19,444)		Women (*n* = 34,384)		Adjusted ORs and 95% CI
	Low-BF (*n* = 16,146)	High-BF (*n* = 3298)	Low-BF (*n* = 27,180)	High-BF (*n* = 7204)	
Height (cm) ^1^	169.0 ± 0.04 ^b^	167.1 ± 0.09 ^a^	157.0 ± 0.03 ^d^	154.9 ± 0.07 ^c^***^+++#^	3.093 (2.881–3.320)
BMI (mg/kg^2^) ^2^	23.9 ± 0.02 ^c^	26.9 ± 0.05 ^a^	22.9 ± 0.02 ^d^	26.1 ± 0.03 ^b^***^###+++^	18.05 (17.03–19.14)
Waist (cm) ^3^	84.3 ± 0.06 ^c^	91.7 ± 0.13 ^a^	76.7 ± 0.05 ^d^	83.4 ± 0.09 ^b^***^###+++^	5.038 (4.794 5.293)
SMI (%) ^4^	7.84 ± 0.01 ^a^	7.50 ± 0.01 ^b^	7.05 ± 0.003 ^c^	6.68 ± 0.006 ^d^***^###+++^	0.858 (0.745–0.954)
Fat mass (%) ^5^	22.4 ± 0.05 ^d^	26.3 ± 0.09 ^c^	29.8 ± 0.04 ^b^	33.9 ± 0.06 ^a^***^+++^	
MetS (%) ^6^	2393 (14.2)	1205 (35.1) ^‡‡‡^	2933 (9.65)	1769 (22.1) ^‡‡‡^	6.289 (5.833–6.780)
glucose (mg/dL) ^7^	98.2 ± 0.17 ^b^	99.6 ± 0.37 ^a^	93.2 ± 0.13 ^d^	94.1 ± 0.25 ^c^***^+++^	1.178 (1.103–1.257)
HbA1c (%) ^8^	5.72 ± 0.01 ^b^	5.77 ± 0.01 ^a^	5.70 ± 0.01 ^b^	5.69 ± 0.01 ^b^***^##^	1.454 (1.321–1.601)
Total cholesterol ^9^	190.4 ± 0.31 ^d^	194.9 ± 0.68 ^c^	199.4 ± 0.24 ^b^	207.3 ± 0.46 ^a^***^++^	1.073 (1.001–1.150)
HDL (mg/dL) ^10^	49.2 ± 0.11 ^c^	49.8 ± 0.24 ^c^	55.8 ± 0.08 ^a^	57.6 ± 0.16 ^b^***^+++###^	1.210 (1.135–1.290)
LDL (mg/dL) ^11^	113 ± 0.28 ^b^	114 ± 0.62 ^c^	121 ± 0.22 ^a^	126 ± 0.42 ^a^***^+++###^	1.089 (1.007–1.177)
TG (mg/dL) ^12^	142 ± 0.72 ^b^	158 ± 1.59 ^a^	115 ± 0.57 ^c^	117 ± 1.07 ^c^***^+++###^	1.229 (1.156–1.308)
Hs-CRP (mg/dL) ^13^	0.153 ± 0.004 ^b^	0.187 ± 0.007 ^a^	0.118 ± 0.003 ^c^	0.154 ± 0.005 ^b^***^+++^	1.266 (1.008–1.589)
SBP (mmHg) ^14^	125.0 ± 0.14 ^a^	124.9 ± 0.24 ^a^	121.0 ± 0.10 ^b^	121.4 ± 0.20 ^b^***	1.105 (1.040–1.175)
DBP (mmHg) ^15^	78.1 ± 0.10 ^a^	77.9 ± 0.16 ^a^	74.4 ± 0.07 ^b^	74.6 ± 0.13 ^b^***^#^	1.040 (0.948–1.140)
eGFR (mL/min) ^16^	84.5 ± 0.16 ^b^	83.0 ± 0.26 ^c^	87.2 ± 0.11 ^a^	87.7 ± 0.23 ^a^***^+###^	0.774 (0.687–0.873)
AST (U/L) ^17^	24.6 ± 0.24 ^b^	25.7 ± 0.40 ^a^	23.1 ± 0.16 ^c^	23.2 ± 0.34 ^c^**^+++^	1.425 (1.250–1.624)
ALT(U/L) ^18^	25.1 ± 0.24 ^b^	27.9 ± 0.39 ^a^	20.5 ± 0.16 ^c^	20.9 ± 0.33 ^b^***^+++###^	1.333 (1.227–1.448)

Values represent adjusted means ± standard error for continuous variables and the number and percentages of participants for categorical variables. Adjusted for age, BMI, education, income, energy intake, alcohol intake, smoking, and total activity. High body fat (case) was defined as 25% and 30% of body weight for men and women, respectively. MetS, metabolic syndrome. The cutoff points of the reference for logistic regression with adjusted for age, BMI, education, income, energy intake, alcohol intake, smoking, and total activity. They were as follows: ^1^ <172.5 cm for men and <160 cm for women; ^2^ <25 kg/m^2^ for BMI; ^3^ <90 cm for men and 85 cm for women waist circumferences; ^4^ <29.0% for men and 22.8% for women in skeletal muscle index (SMI; defined as appendicular skeletal muscle mass/weight); ^5^ <25% for men and 30% for women for fat mass; ^6^ metabolic syndrome (MetS); ^7^ <126 mL/dL fasting serum glucose plus diabetic drug intake; ^8^ <6.5% HbA1c plus diabetic drug intake; ^9^ <230 mg/dL plasma total cholesterol concentrations; ^10^ >40 mg/dL for men and 50 mg/dL for women plasma HDL cholesterol; ^11^ <160 mg/dL plasma LDL cholesterol concentrations; ^12^ <150 mg/dL plasma triglyceride (TG) concentrations; ^13^ <0.5 mg/dL serum high-sensitive C-reactive protein (hs-CRP) concentrations; ^14^ <140 mmHg SBP; ^15^ <90 mmHg DBP plus hypertension medication; ^16^ estimated glomerular filtration rate (eGFR) < 70; ^17^ aspartate aminotransferase < 40 U/L; ^18^ alanine aminotransferase <35 U/L. ** Significantly different by genders at *p* < 0.01; *** at *p* < 0.001. ++ Significantly different by fat mass at *p* < 0.01; +++ at *p* < 0.001. # Significant interactions at *p* < 0.05; ## at *p* < 0.01; ### at *p* < 0.001. ^‡‡‡^ Significantly different from the low-fat mass group at *p* < 0.001. ^a,b,c,d^, different letters on the bar indicate significant differences among the groups in Tukey’s test at *p* < 0.05.

**Table 4:** Pathways related to genetic variants for body fat mass.

Pathways	No. of Genes	Beta	Std	*p*-Value	*p*-Value for Bonferroni Correction
GO BP: GO sodium ion transmembrane transport	133	0.323	0.027	0.081	3.18 × 10^−5^
GO BP: GO myoblast proliferation	17	0.693	0.021	0.179	5.41 × 10^−5^
Curated gene sets: Biocarta flumazenil pathway	8	1.208	0.025	0.326	0.000104
GO BP: GO Neuron apoptotic process	224	0.211	0.023	0.058	0.000129
Curated gene sets: Reactome RMTS methylate histone arginine	60	0.387	0.022	0.108	0.000172
GO MF: GO Peptide hormone binding	47	0.488	0.024	0.137	0.000183
GO BP: GO Positive regulation of vascular endothelial cell proliferation	13	0.778	0.020	0.219	0.000193

No., number; GO, gene ontology; BP, biological process; MF, molecular function; Std, standard deviation; Bon, Bonferroni correction.

**Table 5:** The characteristics of the ten genetic variants of genes related to body fat mass in adults using the generalized multifactor dimensionality reduction analysis.

CHR ^1^	SNP ^2^	Location	Mi ^3^	Ma ^4^	OR ^5^	SE ^6^	*p*-Value for OR ^7^	*p*-Value for OR ^8^	Genes	Feature	^9^ MAF	^10^ HWE
1	rs509325	177894591	G	T	1.123	0.0145	1.37 × 10^−15^	8.28 × 10^−4^	*SEC16B*	Intron	0.2845	0.5035
2	rs6545790	25109302	A	G	1.063	0.0133	4.87 × 10^−6^	4.75 × 10^−4^	*ADCY3*	Intron	0.4374	0.4064
2	rs7560575	54142030	C	T	0.9889	0.0670	2.33 × 10^−7^	1.94 × 10^−2^	*PSME4*	Transcript	0.0112	0.2631
4	rs2196476	20270600	G	A	1.097	0.0191	1.26 × 10^−6^	0.0069	*SLIT2*	Intron	0.135	0.4579
11	rs6265	27679916	C	T	0.923	0.0133	2.45 × 10^−10^	0.0048	*BDNF*	Missense	0.4588	0.1481
13	rs587056	98976374	T	C	1.35	0.0636	2.33 × 10^−10^	0.0067	*FARP1*	Intron	0.0106	0.5496
16	rs1421085	53800954	T	C	1.173	0.0197	6.24 × 10^−16^	2.82 × 10^−6^	*FTO*	Transcript	0.1245	0.4604
17	rs35867081	79047278	G	A	1.065	0.0137	3.99 × 10^−6^	0.0052	*BAIAP2*	Transcript	0.3651	0.2094
19	rs60259426	46340832	G	A	0.938	0.0134	1.54 × 10^−7^	0.00072	*SYMPK*	Transcript	0.4202	0.3135
20	rs6089240	60152260	A	G	0.9278	0.0126	1.60 × 10^−8^	5.81 × 10^−4^	*CDH4*	Intron	0.4641	1.0

^1^ Chromosome; ^2^ single-nucleotide polymorphism; ^3^ minor allele; ^4^ major allele; ^5^ odds ratio for body fat mass by GWAS in a city-hospital-based cohort; ^6^ standard error for body fat mass by GWAS in a city-hospital-based cohort; ^7^ *p*-value for OR for body fat mass after adjusting for age, gender, residence area, survey year, daily energy intake, levels of education, and income in a city-hospital-based cohort; ^8^ *p*-value for OR for body fat mass after adjusting for covariates stated above in an Ansan/Ansung cohort; ^9^ minor allele frequency; ^10^ Hardy–Weinberg equilibrium.

**Table 6:** Adjusted odds ratios for fat mass risk by polygenetic risk scores of the three-SNP model (PRS) for gene–gene interaction after covariate adjustments according to the patterns of lifestyles.

	Low-PRS (*n* = 19,686)	Medium-PRS (*n* = 30,513)	High-PRS (*n* = 3629)	Gene–Nutrient Interaction *p*-Value
Low energy ^1^ High energy	1	1.150 (1.081–1.223) 1.102 (1.021–1.189)	1.228 (1.088–1.387) 1.157 (0.997–1.348)	0.0231
Low CHO ^2^ 70 High CHO	1	1.045 (0.893–1.223) 0.991 (0.927–1.060)	1.006 (0.866–1.169) 1.079 (1.014–1.149)	0.3493
Low protein ^3^ 13 High protein	1	1.123 (1.052–1.198) 0.961 (0.881–1.049)	1.255 (1.103–1.427) 1.019 (0.938–1.107)	0.0272
Low fat ^4^ 15 Moderate fat	1	1.000 (0.926–1.080) 0.994 (0.896–1.102)	1.088 (1.013–1.169) 1.028 (0.932–1.133)	0.0664
Low alcohol ^5^ 20 High alcohol	1	1.139 (1.075–1.208) 1.129 (1.050–1.213)	1.218 (1.086–1.366) 1.204 (1.046–1.386)	0.8083
Low KBD ^6^ High KBD	1	0.997 (0.938–1.061) 1.002 (0.929–1.080)	1.067 (1.007–1.130) 1.083 (1.009–1.162)	0.5368
Low PBD ^6^ High PBD	1	1.132 (1.081–1.185) 1.023 (0.961–1.088)	1.258 (1.126–1.406) 1.110 (1.006–1.224)	0.0026
Low WSD ^6^ High WSD	1	0.997 (0.938–1.061) 0.998 (0.927–1.075)	1.067 (1.007–1.130) 1.074 (1.002–1.151)	0.1356
Low RMD ^6^ High RMD	1	1.112 (1.057–1.169) 1.159 (1.096–1.225)	1.197 (1.134–1.263) 1.254 (1.128–1.395)	0.8419
Low exercise ^7^ High exercise	1	1.034 (0.949–1.128) 0.997 (0.938–1.061)	1.103 (1.018–1.196) 1.067 (1.007–1.130)	0.1778
Non-smoke ^8^ Smoke	1	0.997 (0.938–1.061) 1.071 (0.948–1.210)	1.067 (1.007–1.130) 1.041 (0.926–1.169)	0.1328

Values were expressed as odds ratio and 95% confidence intervals. PRS of the three-SNP model (*BDNF*_rs6265, *FTO*_rs1421085, and *SEC16B*_rs509325) was divided into three categories (0–2, 3–4, and ≥5) by three groups as the low, medium, and high groups of the best model of GMDR. The cutoff points of the parameters were defined as follows: ^1^ <estimated energy intake, ^2^ <70% carbohydrate (CHO), ^3^ <13% protein, ^4^ <15% fat, ^5^ <20 g/day alcohol, ^6^ <75th percentile, ^7^ 30 min moderate exercise for 3 times a week, and ^8^ smoking. Multivariate regression models include the main effects, interaction terms of gene and main effects (energy and nutrient intake), and potential confounders, including sex, age, BMI, the status of smoking and drinking, levels of income and education, physical activity, energy intake, and percent intake for carbohydrate and fat. The reference was low PRS. KBD, Korean-style balanced diet; PBD, plant-based diet; WSD, Western-style diet; RMD, rice-main diet.

## Data Availability

The data can be retrieved from the corresponding author upon reasonable request.

## Supplementary Materials

The following supporting information can be downloaded at: https://www.mdpi.com/article/10.3390/nu15041063/s1, Table S1: The characteristics of the ten genetic variants of genes related to fat mass in adults.
