# Supplementary material for: Association of Plant-Based and High-Protein Diets with a Lower Obesity Risk Defined by Fat Mass in Middle-Aged and Elderly Persons with a High Genetic Risk of Obesity
Source: Nutrients. 2023 Feb 20;15(4):1063. doi: 10.3390/nu15041063 (PMC9960655; doi:10.3390/nu15041063)
Supplement: Supplementary file 1 [file nutrients-15-01063-s001.zip › nutrients-2112676-supplementary.pdf]

Table S1. Generalized multifactor dimensionality reduction (GMDR) results of multilocus interaction with genetic variants mainly related to obesity risk defined with fat mass.

GMDR	Adjusted for sex, age, area, education, BMI				Adjusted for sex, age, area, education, BMI, smoking, alcohol, exercise			
Model	TRBA	TEBA	P value	CVC	TRBA	TEBA	P value	CVC
*SEC16B*_rs509325	0.5154	0.5155	0.001	10	0.5154	0.5155	0.001	10
*BDNF*_rs6265<br>*FTO*_rs1421085	0.5189	0.5173	0.001	6	0.5189	0.5173	0.001	6
*SEC16B*_rs509325<br>plus model 2	0.5229	0.5222	0.001	10	0.5229	0.5222	0.001	10
*BAIAP2*_rs35867081<br>plus model 3	0.5265	0.5174	0.001	5	0.5265	0.5174	0.001	5
*ADCY3*_rs6545790<br>*BDNF*_rs6265<br>*SEC16B*_rs509325<br>*BAIAP2*_rs35867081<br>*SYMPK*_rs60259426	0.5337	0.5167	0.001	9	0.5337	0.5167	0.001	9
*CDH4*_rs6089240 plus model 5	0.5477	0.5146	0.001	10	0.5477	0.5146	0.001	10
*FTO*_rs1421085<br>plus model 6	0.5663	0.5128	0.001	10	0.5663	0.5128	0.001	10
*PSME4*_rs7560575<br>plus model 7	0.5899	0.5098	0.0107	10	0.5899	0.5098	0.0107	10
*SLIT2*_rs2196476 plus model 8	0.596	0.509	0.001	8	0.596	0.509	0.001	8
*FARP1*_rs587056<br>plus model 9	0.6019	0.5069	0.0107	10	0.6019	0.5069	0.0107	10

TRBA, trained balanced accuracy; TEBA, test balance accuracy; CVC, cross-validation consistency; P-value for the significance of GMDR model by sign test with and without adjusting for covariates designated in the table. *COX10,* cytochrome C oxidase assembly factor heme A:farnesyltransferase; *AIG1,* androgen induced 1; DLG2, discs large MAGUK scaffold protein 2; *PLXNA4,* plexin A4; *SOX5,* SRY-box transcription factor 5; *IL12A,* interleukin 12A*; PTPRT,* protein tyrosine phosphatase receptor type T; *NMT1,* N-myristoyltransferase 1; *IFT122,* intraflagellar transport 122; *ARID1B,* AT-rich Interaction domain 1B.
